# Supplementary figures and images for: Effects of traditional Chinese medicine in the treatment of patients with central serous chorioretinopathy: A systematic review and meta-analysis
Source: PLoS One. 2024 Jun 21;19(6):e0304972. doi: 10.1371/journal.pone.0304972 (PMC11192357; doi:10.1371/journal.pone.0304972)

**Supplement 3 Egger's regression test (*P*=0.052)**


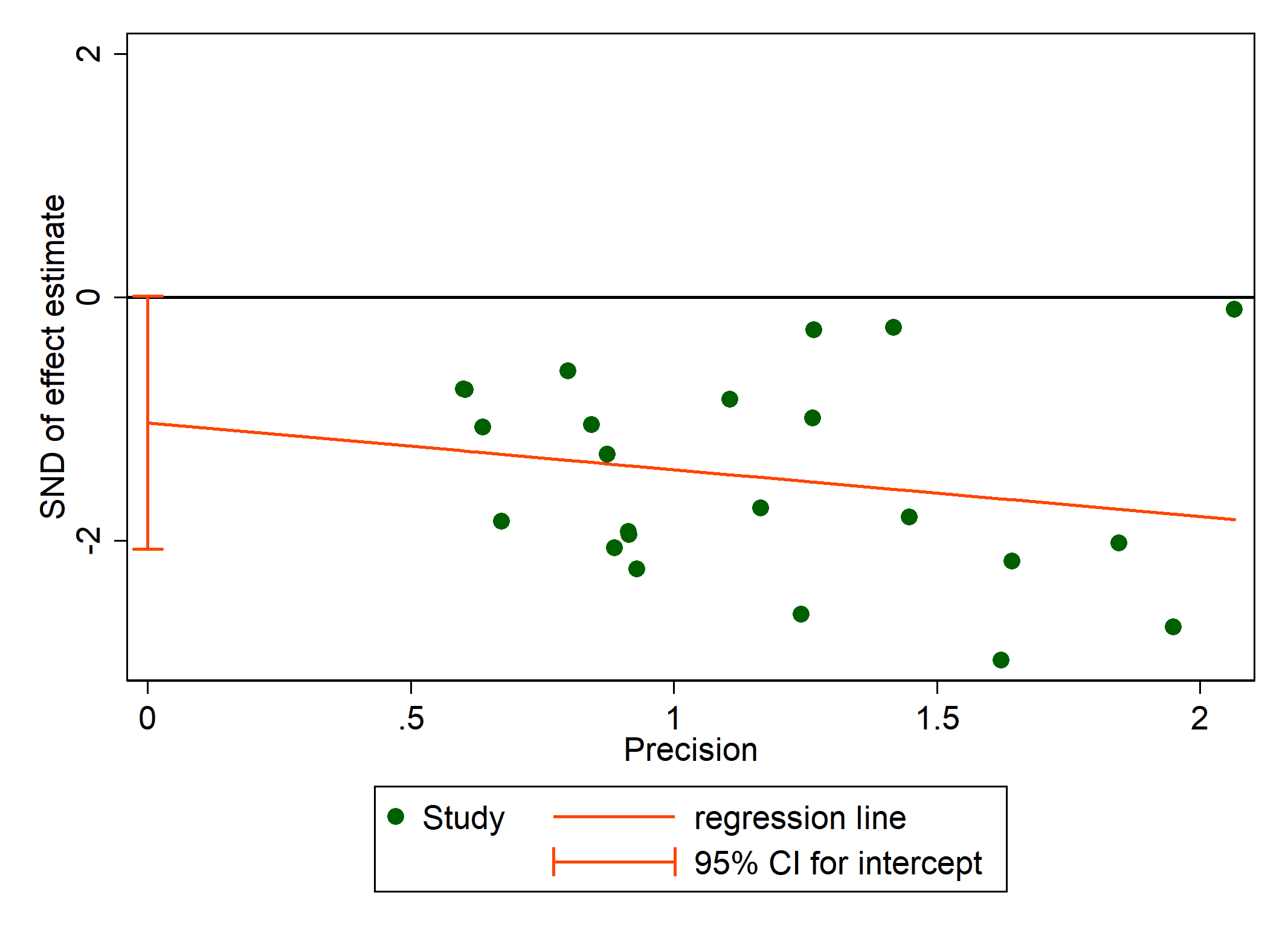


**Egger's regression test based on recurrence rates**

Supplement: S1 Fig — Egger’s regression test based on recurrence rates. (DOC) [file pone.0304972.s001.doc]
