# Supplementary material for: Effects of traditional Chinese medicine in the treatment of patients with central serous chorioretinopathy: A systematic review and meta-analysis
Source: PLoS One. 2024 Jun 21;19(6):e0304972. doi: 10.1371/journal.pone.0304972 (PMC11192357; doi:10.1371/journal.pone.0304972)
Supplement: S1 Table — (DOC) [file pone.0304972.s004.doc]

**Supplement 1 Search strategy**

| No | Database  (Number of articles retrieved) | Search items |
| --- | --- | --- |
| 1 | PUBMED  (n = 0) | 1. "traditional Chinese medicine" [MeSH Terms] 2. “Herbal medicine” OR “Chinese Medicine” OR “Chinese medicine fORmulae” OR “Chinese Herbs” OR “traditional Chinese medicine” OR “herbal therapy” OR “herb therapy” OR “Chinese patent medicine” OR “Chinese herbal drugs” OR “herbal” OR “Chinese patent medicine” OR “Chinese and Western medicine” [All Fields] 3. OR/1,2. 4. "central serous chorioretinopathy" [MeSH Terms] 5. “Central Serous Chorioretinopathies” OR “Chorioretinopathies, Central Serous” OR “Chorioretinopathy, Central Serous” OR “Serous Chorioretinopathies, Central” OR “Serous Chorioretinopathy, Central” OR “Central Serous Retinopathy” OR “Central Serous Retinopathies” OR “Retinopathies, Central Serous” OR “Retinopathy, Central Serous” OR “Serous Retinopathies, Central” OR “Serous Retinopathy, Central” [All Fields]   6. OR/4,5.  7."humans". [MeSH Terms]  8. 3 and 6 and 7. |
| 2 | Cochrane  (n = 0) | (“Herbal medicine” OR “Chinese Medicine” OR “Chinese medicine fORmulae” OR “Chinese Herbs” OR “traditional Chinese medicine” OR “herbal therapy” OR “herb therapy” OR “Chinese patent medicine” OR “Chinese herbal drugs” OR “herbal” OR “Chinese patent medicine” OR “Chinese and Western medicine”) and (“Central Serous Chorioretinopathies” OR “Chorioretinopathies, Central Serous” OR “Chorioretinopathy, Central Serous” OR “Serous Chorioretinopathies, Central” OR “Serous Chorioretinopathy, Central” OR “Central Serous Retinopathy” OR “Central Serous Retinopathies” OR “Retinopathies, Central Serous” OR “Retinopathy, Central Serous” OR “Serous Retinopathies, Central” OR “Serous Retinopathy, Central” OR "central serous chorioretinopathy") |
| 3 | MEDLINE  (n = 0) | 1. randomized controlled trial.pt.  2. controlled clinical trial.pt.  3. randomized.ab.  4. randomly.ab.  5. placebo.ab.  6. drug therapy.ab.  7. trial.ab.  8. groups.ab.  9. OR/1–8.  10. exp animals/ not humans. sh.  11. 9 not 10.  12. ("Case-Control Study" OR "Studies, Case-Control" OR "Study, Case-Control" OR "Case-Comparison Studies" OR "Case Comparison Studies" OR "Case-Comparison Study" OR "Studies, Case-Comparison" OR "Study, Case-Comparison" OR "Case-Compeer Studies" OR "Studies, Case-Compeer" OR "Case-Referrent Studies" OR "Case Referrent Studies" OR "Case-Referrent Study" OR "Studies, Case-Referrent" OR "Study, Case-Referrent" OR "Case-Referent Studies" OR "Case Referent Studies" OR "Case-Referent Study" OR "Studies, Case-Referent" OR "Study, Case-Referent" OR "Case-Base Studies" OR "Case Base Studie" OR "Studies, Case-Base" OR "Case Control Studies" OR "Case Control Study" OR "Studies, Case Control" OR "Study, Case Control" OR "Nested Case-Control Studies" OR "Case-Control Studies, Nested" OR "Case-Control Study, Nested" OR "Nested Case Control Studies" OR "Studies, Nested Case-Control" OR "Study, Nested Case-Control" OR "Matched Case-Control Studies" OR "Case-Control Studies, Matched" OR "Case-Control Study, Matched" OR "Matched Case Control Studies" OR "Matched Case-Control Study" OR "Studies, Matched Case-Control" OR "Study, Matched Case-Control" ).pt.  13. observational study.pt.  14. OR/11–13.  15. (“traditional Chinese medicine” OR “Herbal medicine” OR “Chinese Medicine” OR “Chinese medicine formulae” OR “Chinese Herbs” OR “herbal therapy” OR “herb therapy” OR “Chinese patent medicine” OR “Chinese herbal drugs” OR “herbal” OR “Chinese patent medicine” OR “Chinese and Western medicine”).ab.  16. ("central serous chorioretinopathy" OR “Central Serous Chorioretinopathies” OR “Chorioretinopathies, Central Serous” OR “Chorioretinopathy, Central Serous” OR “Serous Chorioretinopathies, Central” OR “Serous Chorioretinopathy, Central” OR “Central Serous Retinopathy” OR “Central Serous Retinopathies” OR “Retinopathies, Central Serous” OR “Retinopathy, Central Serous” OR “Serous Retinopathies, Central” OR “Serous Retinopathy, Central”).ab.  17. 14 and 15 and 16. |
| 4 | Embase  (n =0) | (central serous chorioretinopathy, traditional Chinese medicine, Herbal medicine, Chinese patent medicine) using a combination of multi-fieldsearch in all fields and EMTREE |
| 5 | China National Knowledge Infrastructure  (n = 425) | ("Chinese Medicine" or "Herbal medicine" or "Traditional Chinese Medicine") [MESH] and ("Central Serous chorioretinopathy") [MESH] |
| 6 | SINOMED  (n = 516) | 1. ("Chinese Medicine" or "Herbal medicine" or "Traditional Chinese Medicine"). [Common field]   2. ("Central Serous chorioretinopathy"). [Common field]  3. 1 and 2. |
| 7 | Wanfang  (n = 430) | 1. ("Chinese Medicine" or "Herbal medicine" or "Traditional Chinese Medicine"). [Common field]   2. ("Central Serous chorioretinopathy"). [Common field]  3. 1 and 2. |
| 8 | Technology Periodical Database (VIP)  (n = 455) | 1.("Chinese Medicine" or "Herbal medicine" or "Traditional Chinese Medicine"). [MESH or keywords]  2.("Central Serous chorioretinopathy"). [MESH or keywords]  3.1 and 2. |
| 9 | [www.clinicaltrials.gov](http://www.clinicaltrials.gov/)  (n = 0) | ("Traditional Chinese Medicine" AND "Central Serous chorioretinopathy" ) |
| 10 | [www.clinicaltrialsregister.eu](http://www.clinicaltrialsregister.eu/)  (n = 0) | ("Traditional Chinese Medicine" AND "Central Serous chorioretinopathy" ) |
| 11 | trialsearch.who.int  (n = 0) | ("Traditional Chinese Medicine" AND "Central Serous chorioretinopathy" ) |
