# Supplementary material for: Effects of traditional Chinese medicine in the treatment of patients with central serous chorioretinopathy: A systematic review and meta-analysis
Source: PLoS One. 2024 Jun 21;19(6):e0304972. doi: 10.1371/journal.pone.0304972 (PMC11192357; doi:10.1371/journal.pone.0304972)
Supplement: S2 Table — (DOC) [file pone.0304972.s005.doc]

**Supplement 2 Evaluation criteria for the overall risk of bias in randomized controlled trials**

| Global rating | Items with high risk | Items with unlcear risk |
| --- | --- | --- |
| Low risk | 0 | =< 3 |
| moderate | 0 | > 3 |
| moderate | 1 | any |
| High risk | >1 | any |
